# Adapting to the projected epidemics of Fusarium head blight of wheat in Korea under climate change scenarios

**DOI:** 10.3389/fpls.2022.1040752

**Published:** 2022-12-09

**Authors:** Jin-Yong Jung, Jin-Hee Kim, Minju Baek, Chuloh Cho, Jaepil Cho, Junhwan Kim, Willingthon Pavan, Kwang-Hyung Kim

**Affiliations:** ^1^ Department of Agricultural Biotechnology, Seoul National University, Seoul, South Korea; ^2^ National Center for Agro-Meteorology, Seoul National University, Seoul, South Korea; ^3^ Wheat Research Team, National Institute of Crop Science, Wanju, South Korea; ^4^ Convergence Center for Watershed Management, Integrated Watershed Management Institute, Suwon, South Korea; ^5^ Korea National University of Agriculture and Fisheries, Jeonju, South Korea; ^6^ International Fertilizer Development Center, Muscle Shoals, AL, United States; ^7^ Graduate Program in Applied Computing, University of Passo Fundo, Passo Fundo, RS, Brazil

**Keywords:** wheat, fusarium head blight, shared socioeconomic pathway, CMIP6, climate change, adaptation measure

## Abstract

Fusarium head blight (FHB) of wheat, mainly caused by *Fusarium graminearum* Schwabe, is an emerging threat to wheat production in Korea under a changing climate. The disease occurrence and accumulation of associated trichothecene mycotoxins in wheat kernels strongly coincide with warm and wet environments during flowering. Recently, the International Panel for Climate Change released the 6th Coupled Model Intercomparison Project (CMIP6) climate change scenarios with shared socioeconomic pathways (SSPs). In this study, we adopted GIBSIM, an existing mechanistic model developed in Brazil to estimate the risk infection index of wheat FHB, to simulate the potential FHB epidemics in Korea using the SSP245 and SSP585 scenarios of CMIP6. The GIBSIM model simulates FHB infection risk from airborne inoculum density and infection frequency using temperature, precipitation, and relative humidity during the flowering period. First, wheat heading dates, during which GIBSIM runs, were predicted over suitable areas of winter wheat cultivation using a crop development rate model for wheat phenology and downscaled SSP scenarios. Second, an integrated model combining all results of wheat suitability, heading dates, and FHB infection risks from the SSP scenarios showed a gradual increase in FHB epidemics towards 2100, with different temporal and spatial patterns of varying magnitudes depending on the scenarios. These results indicate that proactive management strategies need to be seriously considered in the near future to minimize the potential impacts of the FHB epidemic under climate change in Korea. Therefore, available wheat cultivars with early or late heading dates were used in the model simulations as a realistic adaptation measure. As a result, wheat cultivars with early heading dates showed significant decreases in FHB epidemics in future periods, emphasizing the importance of effective adaptation measures against the projected increase in FHB epidemics in Korea under climate change.

## 1 Introduction

Fusarium head blight (FHB), also known as wheat scab, is mainly caused by *Fusarium graminearum* Schwabe and is a deadly fungal disease affecting staple crops, such as wheat, barley, and rice, in many countries (Bai and Shaner, 2004; Kim Y et al., 2018). Under favorable conditions for disease occurrence, FHB deteriorates crop quality and reduces yield, resulting in the economic loss (Parry et al., 1995). Grains infected with FHB can also be contaminated with various mycotoxins produced by the fungal pathogen, posing a health risk to humans and animals (Salgado et al., 2014). Therefore, FHB outbreaks can cause serious socioeconomic disruption. Previous studies report that FHB caused economic losses worth approximately $2.7 billion in the United States over the 20 years since the early 1990s (McMullen et al., 2012). Further, wheat- and barley-growing regions of South America reported a similar trend of increased FHB epidemic frequency and resultant losses in the 1990s (Del Ponte et al., 2009; Moschini et al., 2013; Duffeck et al., 2020). Fusarium head blight also poses a threat to wheat and barley production in many other countries, including China and Canada (Zhu et al., 2019).

Fusarium head blight is a major wheat disease in Korea (Ryu and Lee, 1990; Shin et al., 2018). In South Korea, wheat is cultivated mainly in the southern region; however, it is gradually expanding to the northern provinces, possibly owing to the recent warming climate. In 1963, the first major FHB outbreak devastated the southern region of the country, reducing the wheat yield by 40–80%. Since then, regular outbreaks have been reported approximately every ten years (Chung, 1975). However, owing to abnormal weather patterns and the expansion of wheat cultivation areas, the frequency of the disease outbreak has increased in the last two decades (Park et al., 2012). While severe outbreaks in 2002 resulted in up to 59% of the FHB incidence, the incidence in the southern provinces varied over the years, ranging from 0.1% to 16% (Shim and Gang, 2018), indicating that the occurrence of FHB is indeed affected by the annual variation in weather conditions during critical crop growth stages, including heading and flowering.

The wheat FHB causes distinct symptoms, such as premature bleaching of spikelets or blank heads and accumulation of mycotoxins, such as deoxynivalenol (DON), nivalenol (NIV), and zearalenone (ZEA), that have adverse effects on humans and animals. The FHB pathogen infects wheat anthers through wind dispersal or rain-splash during the flowering period from mid-April to mid-May. Since FHB is a monocyclic disease, the quantity of the primary inoculum is a key factor influencing its incidence. Overwintering pathogens on the crop residue are the main inoculum sources causing new infections during the following spring. Airborne ascospores, produced outside the field, could initiate disease by traveling long distances (Köhl et al., 2007). The FHB infection is greatly affected by weather conditions. During the flowering period, if the environmental conditions are optimal (16 to 30°C along with > 95% relative humidity for 2–3 days), the disease can spread rapidly (Parry et al., 1995). Owing to these epidemiological characteristics, FHB is considered a major plant disease influenced by abnormal weather events under climate change conditions.

A climate change impact assessment study of the FHB epidemic was conducted in Scotland (Skelsey and Newton, 2015). Decreasing risks of FHB epidemics with both limited and non-limited primary inocula were assessed for the 2040s and the 2080s. Similarly, Boland et al. (2004) estimated a possible reduction of FHB epidemics caused by the decreased rates of disease progression due to a projected decrease in rain and leaf wetness in Ontario, Canada. However, a contrary projection was made in Sweden (Roos et al., 2011), showing an increase in mycotoxin contamination owing to a more humid climate in the future. Another study in China simulated the projected increases of FHB epidemics in wheat by inputting the estimated anthesis dates and climate change scenario data into an FHB forecasting model (Zhang et al., 2014). Moschini et al. (2013) also showed an increase in the number of years with moderate or severe FHB incidence under the future climate change scenario in Argentina. These contrasting assessment studies indicate that climate change has complex impacts on FHB depending on the region and thus demand sophisticated impact assessments in future studies (Juroszek and von Tiedemann, 2013).

To conduct a climate change impact assessment on FHB, an ecophysiological model that considers environmental conditions affecting FHB infection and transmission can be used. Several models simulating the epidemics of FHB have been developed for Argentina (Moschini and Fortugno, 1996), Belgium (Detrixhe et al., 2003), Canada (Hooker et al., 2002), Italy (Rossi et al., 2003), the United States (De Wolf et al., 2003; Shah et al., 2013), and China (Xiao et al., 2020). Moschini and Fortugno (1996) developed empirical equations to predict FHB incidence in Argentina using accumulated degree-days and two-day window values of precipitation and relative humidity variables. Detrixhe et al. (2003) developed an ecophysiological model of FHB for winter wheat in Belgium, which predicts FHB infection based on the interpolation of weather radar data and uses the estimation of leaf wetness duration instead of relative humidity. In Canada, three equations using rainfall and temperature data as input variables for 4–7 days before heading were developed to predict the production of DON in mature wheat grains (Hooker et al., 2002). Rossi et al. (2003) developed a dynamic simulation model for FHB infection in wheat in Italy. This model calculates two daily indices, the infection risk of FHB and the mycotoxin content of kernels, based on a systems analysis that includes factors such as sporulation, spore dispersal, and infection of the host tissue (Leffelaar and Ferrari, 1989). In the United States, logistic regression models were developed using weather variables for seven days prior and ten days post anthesis (De Wolf et al., 2003). Other logistic regression models using weather variables before and after anthesis have also been developed in the US (Shah et al., 2013). Xiao et al. (2020) used a dynamic remote sensing technique to predict FHB infections in China.

Among existing FHB models, we used the GIBSIM to simulate the potential epidemics of FHB in wheat in Korea, as it better reflects the environmental conditions affecting the development of FHB during the flowering period (Del Ponte et al., 2005). The GIBSIM model was first developed in Brazil and used as a web-based FHB forecasting system (Fernandes et al., 2007) and a climate variability impact assessment of FHB in southern Brazil (Del Ponte et al., 2009). The GIBSIM model calculates FHB infection risk by combining the effects of multiple epidemiological factors, such as the host, inoculum, and environment. It simulates the accumulated infection index (GIB%) by obtaining the proportional value of each epidemiological factor and then multiplying them. The proportion of host factors is the proportion of susceptible tissue obtained by calculating the anthers extruded in the head emerging at the heading date. The proportion of the inoculum factor reflects the inoculum pressure with daily relative humidity and is a dummy variable for consecutive rainy days. Daily precipitation and relative humidity were used to determine the conducive conditions for FHB infection and the proportion of possible infection events.

The GIBSIM model requires information on the wheat heading date as a key input to simulate FHB infection risk. Since the heading date could change due to the increasing temperature under climate change, the potential FHB epidemic can be predicted realistically by integrating the simulation of wheat heading dates into the modeling process with the GIBSIM in this study. Many phenological models, including empirical models based on the accumulation of thermal time and mechanistic models simulating the emission of leaves and spike primordia at the shoot apex, have been used to predict wheat phenology (Bogard et al., 2014). We adopted the development rate (DVR) model introduced by Maruyama et al. (2010) to estimate the wheat heading date for each simulation site and period.

Wheat is not the main staple crop in Korea, and most of the wheat consumed in Korea is imported from the United States, Canada, and Australia (Statistics Korea, 2022). Therefore, the self-sufficiency rate of wheat production in South Korea is at a meager 0.5%. However, owing to food security issues due to the recent COVID-19 pandemic and international conflicts limiting the trade of food and natural resources, the importance of wheat production in Korea is increasing. In addition, considering the recurring food crisis due to rapid global and climate change and the modest self-sufficiency rate (20.2% as of 2020) of grain crops in South Korea, the government has been trying to increase domestic wheat production by implementing the “wheat industry development basic plan” policy aimed at achieving a 10% wheat self-sufficiency rate by 2030. Wheat is currently cultivated only in the southern region of South Korea. Wheat cultivation areas, benefiting from government support and the warming winter temperatures, are likely to expand northward. Therefore, assessing the projected impacts of climate change on the FHB epidemic in Korea is necessary. In this study, we determined the areas suitable for wheat cultivation at present and in the future based on the guidelines of the Rural Development Administration (RDA) of South Korea, predicted wheat heading dates for each area using the DVR model, and then simulated the future FHB epidemics using the GIBSIM over the suitable areas and during the predicted heading periods from the respective models. Finally, we examined the projected FHB epidemics in alternative wheat cultivars with early or late heading dates as an adaptation strategy to climate change.

## 2 Materials and methods

### 2.1 Study area and climate data

**Figure 1:**
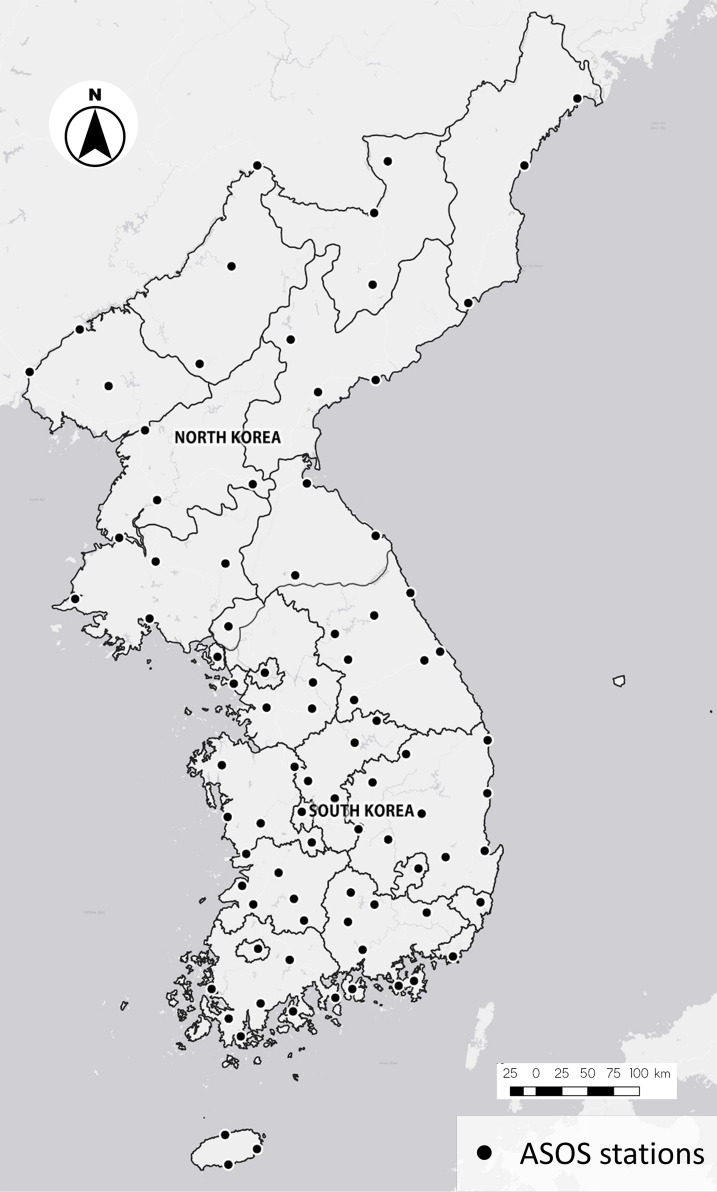
The Korean Peninsula with the location information of the 87 Automatic Synoptic Observation System (ASOS) stations used in the study.

The target area of this study was the entire Korean Peninsula (latitude 37°31’ N, longitude 127°01’ E), including both South and North Korea (**Figure 1**). The spatial variability in climate over the peninsula is influenced by topography, consisting of mountains (57.4%) and plains (35.1%). In addition, the climate differs dramatically from north to south, spanning approximately 11 degrees of latitude (approximately 1100 km distance). South Korea experiences a relatively warm and wet climate affected by the warm East Korea Warm Current, whereas North Korea experiences a colder and, to some extent, an inland climate similar to that of the continent. A recent climate change assessment report published by the Korea Meteorological Administration (KMA) showed that the historical records of air temperature over the Korean Peninsula show a faster rise than the global mean trend. Further, the seasonal precipitation variability considerably increased over the past few years (Korea Meteorological Administration, 2020).

Daily weather data, such as maximum and minimum air temperature (°C), precipitation (mm), relative humidity (%), and solar radiation (W m^-2^), from 1981 to 2021, were obtained from 87 Automatic Synoptic Observation System (ASOS) stations. These stations are evenly distributed over the Korean Peninsula (**Figure 1**), representing most of the local climate of the peninsula. The ASOS data are quality-controlled by the KMA and can be downloaded from the Open Meteorological Data Portal (https://data.kma.go.kr). In this study, the ASOS weather data for 1981–2010 were used as the observation weather data (hereafter, the observation data) for bias-correction of or comparison with climate change scenario data (hereafter, the scenario data) from global climate models (GCM). Owing to considerable missing solar radiation data in the ASOS data, we used daily climatological averages of solar radiation from 1993 to 2021, obtained from the National Aeronautics and Space Administration–Prediction Of Worldwide Energy Resources (NASA–POWER), for each ASOS station (Sayago et al., 2020).

The Shared Socioeconomic Pathways (SSP) scenario data from the sixth phase of the Coupled Model Intercomparison Project (CMIP6) by the Intergovernmental Panel on Climate Change (IPCC) were used in this study. The SSP scenarios include new social and economic factors along with the Representative Concentration Pathway (RCP), thus allowing the projection of future changes in energy and land use based on adaptation and mitigation scenarios (O’Neill et al., 2017; Wang et al., 2018). The SSP scenarios are divided into five main groups (SSP119, SSP126, SSP245, SSP370, and SSP585) based on future mitigation and adaptation efforts considering future socioeconomic changes and various radiative forcings in the Fifth Assessment Report (AR5). Among them, two scenarios, SSP245 and SSP585, were used as future climate change scenarios in this study. As the current emissions are comparable to the RCP8.5 pathway (Schwalm et al., 2020), these two scenarios are thought to be the most relevant for policymakers to develop policy interventions for climate change in Korea (Lee et al., 2022b). For example, SSP245 assumes a scenario in which a range of technologies and strategies for reducing greenhouse gas emissions are employed in Korea, resulting in stabilized anthropogenic radiative forcing at 4.5 W m^-2^ in 2100. If the “net-zero” emissions or carbon neutrality policy are realized, as pledged by the South Korean government in 2020 (Ministry of Environment, http://eng.me.go.kr), the SSP245 scenario is highly likely to occur in the future. On the contrary, SSP585 corresponds to a nominal anthropogenic forcing of 8.5 W m^-2^ by 2100, assuming a continuously increasing trend of greenhouse gas emissions owing to limited global/national policy intervention in the future (Olivier et al., 2017).

CMIP6 scenario data are available from the Earth System Grid Federation (Williams et al., 2016). In this study, daily weather variables of maximum and minimum air temperatures, relative humidity, precipitation, and solar radiation from 1981 to 2100 were collected from 18 GCMs for two scenarios: SSP245 and SSP585 (**Table 1**). For the subsequent model simulation, the scenario data from 18 GCMs were divided into three periods:1981–2010, 2041–2070, and 2071–2100. Scenario data from 1981 to 2010 were used to represent the historical period, whereas scenario data for 2041–2070 were used for the near future period and 2071–2100 for the distant future period.

**Table 1:** 18 GCMs for the CMIP6 climate change scenarios.

Model	Origin	Country	Resolution	Reference
GFDL-ESM4	Geophysical Fluid Dynamics Laboratory	USA	360 * 180	(John et al., 2018)
MRI-ESM2-0	Meteorological Research Institute	Japan	320 * 160	(Yukimoto et al., 2019)
CNRM-CM6-1	Centre National de Recherches Meteorologiques	France	24572 grids over 128 latitude circles	(Voldoire, 2019)
CNRM-EMS2-1				(Seferian, 2019)
IPSL-CM6A-LR	Institute Pierre-Simon Laplace	France	144 * 143	(Boucher et al., 2019)
MPI-ESM1-2-HR	Max Planck Institute for Meteorology	Germany	384 * 192	(Schupfner et al., 2019)
MPI-ESM1-2-LR			192 * 96	(Wieners et al., 2019)
UKESM1-0-LL	Met Office Hadley Centre	UK	192 * 144	(Good et al., 2019)
ACCESS-CM2	Commonwealth Scientific and Industrial Research Organisation, Australian Research Council Centre of Excellence for Climate System Science	Australia	192 * 144	(Dix et al., 2019)
ACCESS-ESM1-5	Commonwealth Scientific and Industrial Research Organisation	Australia	192 * 145	(Ziehn et al., 2019)
CanESM5	Canadian Centre for Climate Modelling and Analysis	Canada	128 * 64	(Swart et al., 2019)
INM-CM4-8	Institute for Numerical Mathematics	Russia	180 * 120	(Volodin et al., 2019a)
INM-CM5-0			180 * 120	(Volodin et al., 2019b)
EC-Earth3	EC-Earth-Consortium		512 * 256	EC-Earth Consortium (EC-Earth) (2019)
MIROC6	Japan Agency for Marine-Earth Science and Technology, Atmosphere and Ocean Research Institute, National Institute for Environmental Studies, RIKEN Center for Computational Science	Japan	256 * 128	(Shiogama et al., 2019)
MIROC-ES2L			128 * 64	(Tachiiri et al., 2019)
NorESM2-LM	NorESM Climate modeling Consortium consisting of CICERO	Norway	144 * 96	(Seland et al., 2019)
KACE-1-0-G	National Institute of Meteorological Sciences, Korea Meteorological Administration	Korea	192 * 144	(Byun et al., 2019)

The scenario data obtained from the GCMs deviated significantly from the observation data obtained from the ASOS stations. Therefore, using them directly as inputs in impact modeling is challenging. In particular, agricultural impact models, such as crop growth, phenology, and pest and disease models, are highly sensitive to systemic bias in scenario data from GCMs (Ines and Hansen, 2006; Laux et al., 2021). The simple quantile mapping (SQM) method was used for bias correction in this study. SQM is a non-parametric bias correction method that uses empirical quantile mapping to estimate the bias between the observation data for each quantile and the scenario data from GCMs. It is capable of minimizing the overestimation that may be caused by the theoretical cumulative distribution function (CDF) equation. To conduct bias correction, we used 30 years of observation data for 1981–2010 obtained from the ASOS stations as a reference for the SQM. The scenario data from 18 GCMs were bias-corrected and spatially downscaled to 87 weather station points for three simulation periods (historical, near future, and distant future) using the rSQM package of the R programming language (R version 4.2.1) (Cho et al., 2018). More details on the SQM implementation for the bias correction of GCM data are available in previous studies by Cho et al. (2020) and Lee et al., (2022b).

The reproducibility of the bias-corrected and downscaled CMIP6 scenarios from 18 GCMs was evaluated based on spatial comparisons between the observation and scenario weather data for the historical period (1981–2010). The reproducibility test was conducted for three weather variables (average air temperature, total precipitation, and average relative humidity) in April and May because the flowering of wheat—corresponding to the simulation period of the FHB epidemic—took place during these months of the historical period (1981–2010). Solar radiation was omitted because the NASA–POWER averages were used in the ASOS data. The monthly average values of the corresponding weather variables were compared, and the percentage differences between the scenario and observation data were visualized to examine the reproducibility of the scenario data from the GCMs. Reasonably good agreement for the percent difference maps of all three weather variables is shown in **Supplementary Figure S1**. The reproducibility tests indicate that the bias-corrected and downscaled CMIP6 scenarios are comparable to the observation data, allowing their use for the subsequent analyses of impact assessment and adaptation studies using the DVR model for simulating wheat heading date and GIBSIM for simulating the FHB epidemic.

### 2.2 Models

#### 2.2.1 The DVR model to predict the heading date of wheat

The developmental rate (DVR) model, introduced by Maruyama et al. (2010), was used to predict the heading date of the Geumgang cultivar for winter wheat. The Geumgang cultivar is cultivated in over 70% of wheat areas in South Korea. The DVR model estimates phenological development based on the numerical relationship between DVR and daily weather data and has been widely used for several crops (Sameshima, 2000; Maruyama et al., 2010; Zhang and Tao, 2013). The DVR model can predict major growth stages relatively accurately and with lesser effort, according to the weather information of the crop growth period. The phenological stage of wheat is represented by the developmental index (DVI). Starting from the sowing date (DVI = 0), the point at which the accumulated DVR value reached 1 was considered the heading date (DVI = 1) (Eq. 1).


(1) $$DVI_{n}=\sum_{i=1}^{n}DVR_{i}$$

where *DVI_n_* is the developmental index on day *n*, and *DVR_i_* is the developmental rate on day *i*.

The DVR values were calculated using Eq. (2), which requires two weather variables, the daily mean air temperature (*T*) and daily photoperiod (*L*), and five parameters, including the minimum number of days from emergence to heading (*G_v_*), temperature when the DVR value becomes half of the maximum rate (*T_hv_*), critical photoperiod (*L_c_*), temperature parameter (*A_v_*), and photoperiod parameter (*B*). Further information on the definition and response of the model parameters is described in detail by Maruyama et al. (2010). Kim et al. (2022) determined five parameters using the observed heading dates of the Geumgang cultivar collected from eight distinct agroclimatic sites in Korea from 2011 to 2021.


(2) $$\begin{array}{ccc} DVR & =\frac{1}{G_{v}}\times \frac{1-exp\left\{-B\left(L-L_{c}\right)\right\}}{1+exp\left\{-A_{v}\left(T-T_{hv}\right)\right\}},\; & for\;B\left(L-L_{c}\right)\geq 0\\ & =\;0, & for\;B\left(L-L_{c}\right)\geq 0 \end{array}$$

The air temperature variable (*T*) was obtained either from the ASOS data or from the CMIP6 scenario data of the 18 GCMs used in the study, and the photoperiod variable (*L*) was estimated using Eq. (3) (Allen et al., 1998)


(3) $$L=\frac{24}{\pi }\left[{\text{cos}}^{-1}\left(-\text{tan }\phi \text{ tan}\delta \right)\right]$$

where *ϕ* is the latitude, and *δ* is the declination of the sun.

The average prediction errors for the estimated heading date using the DVR model were as follows: ME = 0.9, RMSE = 5.1 (day), and the coefficient of determination (R^2^) = 0.56. In the study, with the DVR model, heading dates of the Geumgang cultivar were estimated for all 87 ASOS stations using either the observation data from the ASOS stations or scenario data from the 18 GCMs.

Suitable areas for winter wheat were determined based on official guidelines published by the RDA in Korea (Rural Development Administration, 2020). The RDA guideline instructs that winter wheat can grow where the daily minimum temperature in January ranges from −9 °C to 10 °C. Therefore, the suitable areas were determined as follows: the ASOS observation data and the GCM scenario data were divided into decadal (10-year) periods, and decadal averages of daily minimum temperature in January were calculated and used for the determination of suitable areas in every decadal interval. The sowing date was predicted only for the selected suitable areas by using the quadratic equations in Eq. (4) and Eq. (5), based on the relationship between the average minimum temperature in January and the optimal sowing date for each area under distinct elevations, either below or above 100 m from the sea surface.


(4) $$\begin{array}{cc} \left[Below\;100m\right] & \;y=-0.1017x^{2}+2.2899x+305.98 \end{array}$$

(5) $$\left[More\;than\;100m\right]\;y=\;\;-0.081x^{2}+2.2603x+299.35$$

where *x* is the daily minimum temperature in January averaged over 10 years, and *y* is the estimated sowing date of winter wheat.

As a result, the sowing dates of the Geumgang cultivar for the 87 ASOS stations were calculated for the historical (1981–2010) and two future periods (2041–2070 and 2071–2100) using the ASOS observation data and the scenario data of two SSP245 and SSP585 scenarios from 18 GCMs. Using the same weather input data and DVR model (Eq. 1, 2, and 3), heading dates were predicted from the estimated sowing dates.

#### 2.2.2 The GIBSIM model used to predict the FHB epidemics

The GIBSIM, an FHB infection risk model (Del Ponte et al., 2005), was used to simulate the FHB epidemics at the 87 ASOS stations. Daily weather variables, average air temperature, total precipitation, and average relative humidity were used as input data for the model simulation.

The GIBSIM was first developed and successfully used for FHB early warning and climate variability studies in Brazil (Del Ponte et al., 2005; Fernandes et al., 2007; Del Ponte et al., 2009). The model considers three epidemiological factors related to FHB infection: host, inoculum, and environmental factors. The final output of the model is the accumulated infection index (GIB%; hereafter, FHB risk index) as a function of the proportion of susceptible tissue (ST), infection frequency (INF), and FHB spore cloud density (GZ). Individual equations to determine the level of contributions from each epidemiological factor are available in detail in the study by Del Ponte et al. (2005). In addition, **Supplementary Figure S2** also provides the diagram for the modeling structure of the GIBSIM, adopted from **Figure 1** in Del Ponte et al. (2005), and the details of simulation mechanisms and equations used in the study.

Del Ponte et al. (2005) estimated disease incidence values using linear regression adjusted to the observed FHB incidence data collected in the experimental fields in Brazil. Similarly, to estimate FHB incidence from the FHB risk index of GIBSIM in Korea, we fitted a linear regression model between the FHB risk index and the actual FHB incidence in Korea. For this, the observed FHB incidence data (N=52) collected from major wheat and barley fields in Korea for 2015–2021 were used, and the GIBSIM was run to generate the corresponding FHB risk index using the observed data from the nearest ASOS stations for individual FHB survey data. The linear regression analysis using the observed FHB incidence data and the simulated FHB risk index resulted in a regression equation of *y* = 4.06 *x* + 1.19 (*y*: FHB incidence; *x*: FHB risk index) and an R^2^ of 0.55 (**Supplementary Figure S3**). This equation was used to estimate the ballpark figure of disease incidences from the FHB risk indices of GIBSIM throughout the study. This is because the risk index is a theoretical value for FHB infection risk, which hinders the readers from intuitively understanding the magnitudes of climate change impact and adaptation assessment results in the study.

### 2.3 Climate change impact and adaptation assessments

We used the GIBSIM to simulate FHB epidemics in the Korean Peninsula using *in situ* observations (the observation data) from 87 ASOS stations and climate change scenarios (the scenario data) from 18 GCMs of the CMIP6. Daily weather variables, such as maximum air temperature, minimum air temperature, total precipitation, average relative humidity, and average solar radiation, were used as input data for the model simulation. The predicted heading dates from the DVR model were used to initiate the GIBSIM simulations for each individual season. All GIBSIM simulations were run from the initial heading date (five days before the predicted heading date) to the date when susceptible tissues no longer existed, indicating that the simulation duration for each year was automatically determined in the model. The average duration of the model simulation was very similar for all periods: for example, 38.7, 38.3, and 39.4 days for the historical, near future, and distant future periods, respectively, in the SSP585 scenario.

To assess the climate change impact on FHB epidemics in Korea, GIBSIM was simulated for 87 ASOS stations based on two emission scenarios (SSP245 and SSP585) of CMIP6. Scenario data from 18 GCMs of the CMIP6 that were bias-corrected using the SQM were used as input data for the model simulation. The resulting simulation results were divided into three periods (1981–2010 for the historical, 2041–2070 for the near future, and 2071–2100 for the distant future). To visualize the results on the map, we created a multi-model ensemble (MME) using 30-year simulations for each period. Briefly, for a given GCM at each ASOS station, the average FHB risk indices over the 30-year GIBSIM simulations were calculated. The resultant one average value per GCM was then averaged for the 18 GCMs for each emission scenario (SSP245 or SSP585) to calculate the MME means. The MMEs of the FHB risk index were then converted into FHB incidences using the regression equation between the simulated FHB risk indices and the actual FHB incidences, shown in the 2.2.2 section. These were then visualized on the maps by spatially interpolating the 87 ASOS point values over the Korean Peninsula using the kriging method (Nelson et al., 1999).

Based on the GIBSIM simulations, we also proposed a possible adaptation strategy using wheat cultivars with different heading dates. The underlying assumption of using alternative cultivars with different heading dates is that early or late heading dates will avoid environmental conditions conducive to FHB infection. Changing planting dates or using cultivars of different maturity to avoid high disease pressure periods is a popular adaptation strategy to mitigate the projected impacts of climate change on plant diseases (Nouri et al., 2017; Kim and Koh, 2019). A series of GIBSIM simulations were run to determine the effect of the changed (early or late) heading date on the FHB risk index. Briefly, the heading dates of the wheat cultivars released in South Korea were collected, and the relative differences to those of Geumgang, a cultivar used in the impact assessment, were used to determine the ranges of heading date adjustment in the GIBSIM simulation. The heading dates of the additional cultivar were ranged from 10 days earlier (−10) to 10 days later (+10), compared to the one of Geumgang. Simulations were conducted by adjusting the predicted heading dates from the DVR model at 5-day intervals (−10, −5, 0, + 5, and +10 days) and using the scenario data from 18 GCMs for future periods (2041–2070 and 2071–2100) of the SSP585 scenario. The simulation results were converted into FHB incidences using the regression equation in the 2.2.2 section and then summarized as the average FHB incidences of the entire Korean Peninsula for each future period.

To investigate whether the differences in the simulated risks from changing heading dates with different wheat cultivars were related to projected changes in each weather variable under climate change, we calculated the mean temperature, total precipitation, number of rainy days with more than 0.3 mm precipitation, and mean relative humidity for the simulation duration of the GIBSIM for each run. The number of rainy days over 0.3 mm was selected as it was used to determine the GZ (the daily relative density of an airborne FHB spore cloud) in the model. We conducted this analysis using only the SSP585 scenario data for the distant future period, as it showed the most considerable differences between different heading dates. The results were plotted using a box plot to compare the distribution of the projected changes in each weather variable, representing non-adaptation (using heading dates of the Geumgang) versus the early (−10 days) and the late (+10 days) heading dates, representing adaptation using different cultivars. In addition, a regression analysis with FHB incidences as a dependent variable and four weather variables as an explanatory variable was conducted to statistically understand the relative contribution of individual weather variables to the simulated FHB incidences.

## 3 Results

### 3.1 Suitable areas for winter wheat cultivation with the predicted heading dates from the DVR model

Prior to the impact assessment of future changes in FHB epidemics, the reproducibility of the essential weather variables (air temperature, precipitation, and relative humidity) from the CMIP6 scenario data was evaluated for April and May, corresponding to the duration of the GIBSIM simulations for the historical period (**Supplementary Figure S1**). With respect to average air temperature, the spatial distribution produced using the observation data showed similar results to the scenario data from 18 GCMs. The difference (%) between the observation and scenario data for the air temperature in April ranged from –9.8 to +8.9% (less than 0.63°C in absolute terms) over the Korean Peninsula. Mountainous areas in North Korea showed relatively higher differences (%) between the observation and scenario temperature data in April, with the maximum difference (–9.8%) in Baekdu-san, the highest mountain in Korea. Other than this specific case (air temperature in April), the reproducibility tests of other variables in both April and May resulted in smaller differences (%). While the air temperature in May ranged from –0.19 to +5.35%, the precipitation in April and May ranged from –0.56 to +1.64% and –0.49 to +3.46%, respectively. Further, the relative humidity in April and May ranged from –1.81 to +0.06% and –1.03 to +0.05%, respectively. The SQM used for the bias correction of the CMIP6 scenario tended to overestimate the precipitation in North Korea while underestimating it in South Korea. In addition, the SQM tended to underestimate the relative humidity throughout the Korean Peninsula, as shown in the difference (%) maps in **Supplementary Figure S1**. Consequently, based on these results, the bias-corrected scenario data from 18 GCMs showed reasonably good reproducibility to be used as input data for the models to simulate the wheat heading date and FHB risk index in the study.

**Figure 2:**
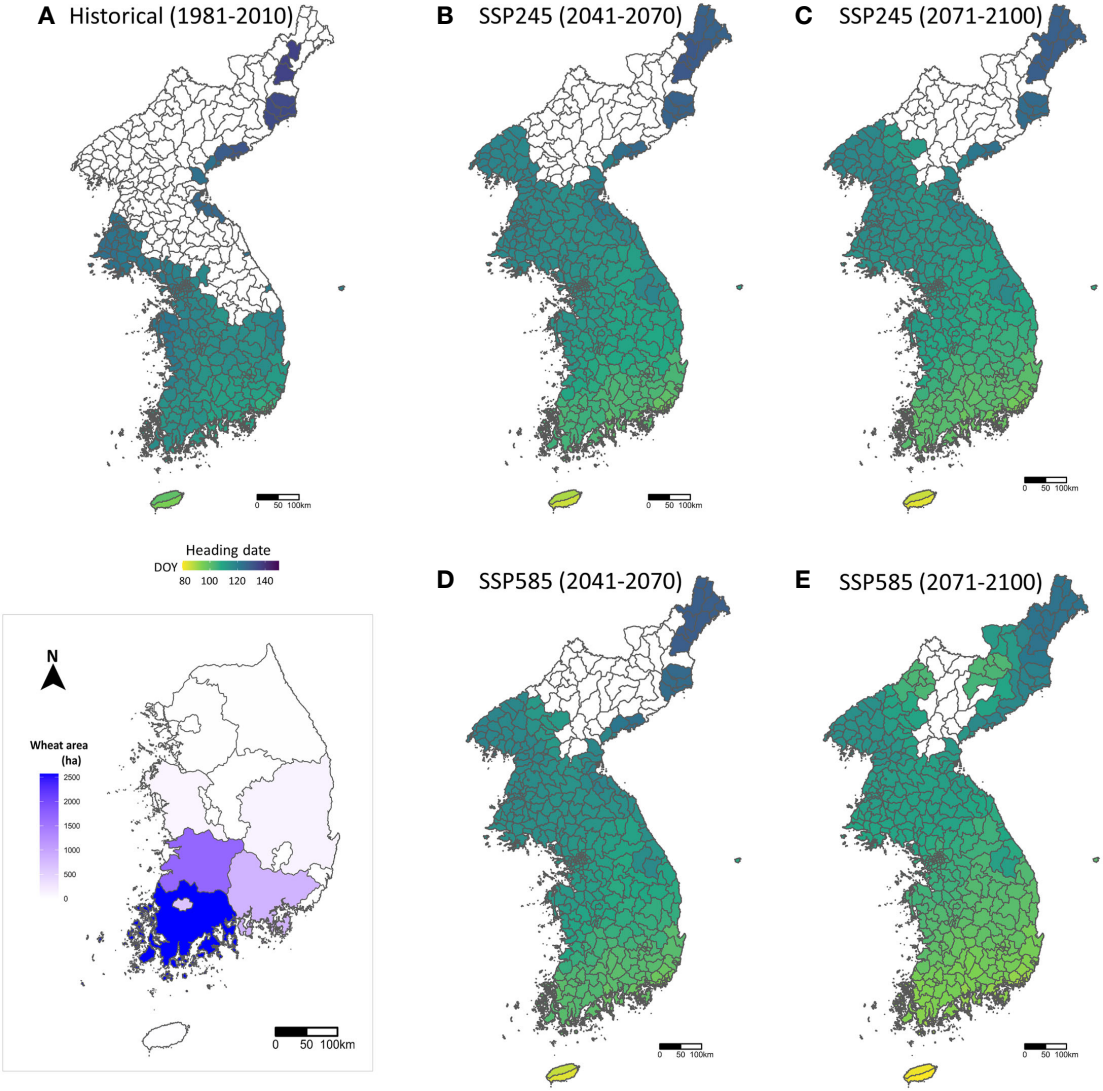
Areas suitable for wheat cultivation and the day of year (DOY) of the predicted heading dates on the maps of the Korean Peninsula in 1981-2010 **(A)** for the historical period, 2041–2070 **(B)** and 2071–2100 **(C)** under the SSP245 scenario, and 2041–2070 **(D)** and 2071–2100 **(E)** under the SSP585 scenario for the future periods. The colored areas indicate suitable wheat cultivation areas, and the DOY of the heading dates is expressed in color. The bottom left map (inset) shows the actual wheat cultivation areas **(ha)** in the provinces of South Korea for 2018–2022, obtained from the Korean Statistical Information Service (https://kosis.kr).

Climatic suitability maps for wheat cultivation in the Korean Peninsula were generated based on the climatic conditions of the SSP245 and SSP585 scenarios for three periods (1981–2010 for historical, 2041–2070 for the near future, and 2071–2100 for the distant future). The projected spatiotemporal changes in the suitable areas for wheat cultivation (uncolored area indicates ‘Not suitable’ and colored area indicates ‘Suitable’) for each administrative area are indicated in **Figure 2**. The simulated suitable areas for the historic period show a good agreement with the actual wheat-growing areas in Korea for 2018–2022 (Korean Statistical Information Service, http://kosis.kr). Despite numerous factors, such as crop rotation, abiotic and biotic stresses, and socioeconomic factors, that affect actual areas for wheat cultivation, these results indicate that the climatic conditions used in this study can be used as a representative of the actual wheat cultivation conditions.

With climate change, the geographical areas that can support wheat cultivation will gradually expand from the present coastal and southern areas to higher inland and northern areas by 2100. In the historical period, more than 50% of the Korean Peninsula appears to be ‘Not suitable’ for wheat cultivation. However, in the distant future, more than 80% of the areas can be marked as ‘Suitable’ (**Figure 2**). This result strengthens the rationale for this study; new environments in the future, either from the expansion of suitable areas or owing to climate change, will introduce new challenges, such as sudden FHB outbreaks, to wheat growers in Korea. Comparing the two SSP scenarios, the suitable areas for wheat cultivation in the near future are similar. In contrast, in the distant future period, wheat cultivation becomes possible in a larger area in the SSP585 scenario than in SSP245 due to an increase in minimum temperature in January.

For areas suitable for wheat cultivation, the heading dates of the Geumgang cultivar were predicted using the DVR model. The day of year (DOY) of the predicted heading dates shown on the maps indicate that the heading dates occur earlier in the south and later in the north, primarily because of the temperature-dependent development rate in phenology development. The average heading date for the historical period is 116 DOY, which, in the distant future period (2071–2100), advances to 108 DOY and 102 DOY in the SSP245 and SSP585 scenarios, respectively. This result suggests that increasing temperatures due to climate change significantly affect the phenological development of wheat in Korea.

### 3.2 Predicting climate change impacts on FHB epidemics using the GIBSIM

**Figure 3:**
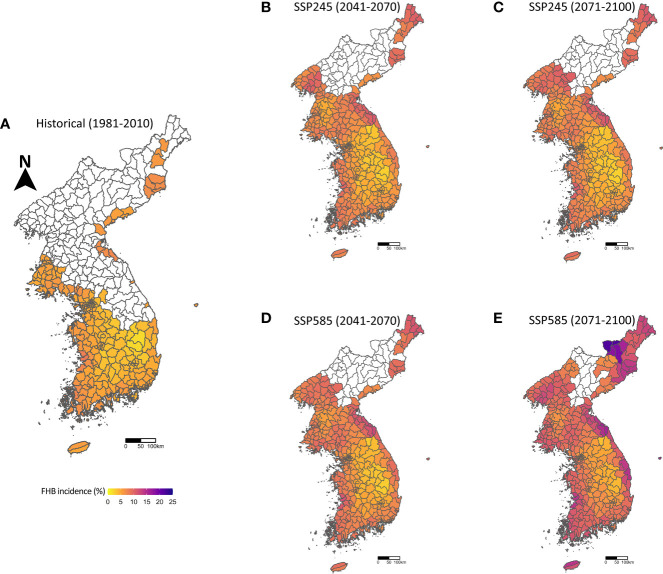
Projected FHB epidemics over the Korean Peninsula in 1981–2010 **(A)** for the historical period, 2041–2070 **(B)** and 2071–2100 **(C)** under the SSP245 scenario, and 2041–2070 **(D)** and 2071–2100 **(E)** under the SSP585 scenario for the future periods. The impact of climate change on FHB incidences was assessed for the suitable wheat cultivation areas using the predicted heading dates, as shown in **Figure 2**.

On suitable wheat cultivation areas and using the predicted heading dates (**Figure 2**), the effects of climate change on the FHB epidemics were assessed based on the SSP245 and SSP585 scenarios, and the MME (30-year average of the GIBSIM simulations using 18 GCM scenario data) of the FHB incidences for individual administrative areas were calculated and interpolated on the maps (**Figure 3**). In the historical period (1981–2010), most areas suitable for wheat showed relatively mild incidences of FHB epidemics, with an average incidence rate of 5.1%. This was consistent with the observed FHB incidence in the field. In fact, the epidemics of FHB in South Korea have been below an economical threshold level, despite varying from 0.1% to 16% since 2002 due to the annual variation in weather conditions in the past years. However, the projected FHB incidences across the Korean Peninsula tended to be more severe towards future periods: 5.1%, 7.2%, and 9.2% of the average FHB incidences for the historical, near future, and distant future periods, respectively, in the SSP585 scenario. Notably, in the SSP245 scenario, a few areas in Korea showed slightly decreased FHB incidences (up to 11.3%) in the distant future period compared to the ones in the near future (data not shown).

Comparing South and North Korea, the incidence of FHB is projected to be relatively higher in North Korea than in South Korea; the average incidences of FHB in South and North Korea in the SSP585 scenario are 6.9% and 7.7%, respectively, for the near future, and 9.0% and 9.5% for the distant future period. These differences indicate that climate conditions in North Korea will become more conducive to FHB epidemics due to climate change. In particular, Hamgyeongbuk-do, the mountainous province in North Korea, showed the highest incidence of FHB, up to 21.8% in the distant future period. In most areas, the FHB incidence was relatively higher in SSP585 than in SSP245. Notably, in the distant future, the incidence of FHB will significantly increase in the coastal areas of the Korean Peninsula.

### 3.3 Adapting to the projected FHB epidemics

A possible adaptation strategy to the projected FHB epidemics was suggested using currently available wheat cultivars, either with earlier or later heading dates than the Geumgang cultivar. The underlying assumption is that advancing or delaying the timing of susceptible stages of wheat may help avoid environmental conditions conducive to FHB infection. Indeed, changing heading dates from −10 to +10 days at 5-day intervals compared to those of the Geumgang cultivar resulted in significant changes in FHB incidences (**Table 2**). The cultivars with an earlier heading date had a lower FHB incidence than the Geumgang cultivar, whereas the cultivars with a later heading date had a higher FHB incidence. Wheat cultivars with 10 days earlier heading dates showed a more substantial reduction in FHB incidences compared to those with 5 days earlier heading dates, despite limited availability for the cultivars with 10 days earlier heading dates, as shown in **Table 2**. Cultivars with early heading dates showed reductions of 17.3% (5 days earlier heading date) and 32.1% (10 days earlier heading date) in the average incidence of FHB in the near future, whereas the extent of risk reduction was decreased in the distant future. In contrast, later heading dates resulted in higher incidences of FHB in the near future: 19.5% with 5 days later heading date and 40.7% with 10 days later heading date. However, no further increase in FHB incidence was simulated in the near future. Instead, slightly decreased FHB incidences were recorded in the near future period. Overall, our adaptation analysis suggested that the Arijinheuk, Baekgang, Hwanggeumal, Joeun, Jogyeong, Johan, Jojung, Jonong, Jopum, and Saeol cultivars could be selected as alternatives for coping with the projected FHB epidemics in the future by replacing the Geumgang, a major wheat cultivar, in South Korea.

**Table 2:** Manipulating the FHB risks for the near future (2041–2070) and the distant future (2071–2100) periods under the SSP585 scenario, through an adaptive measure of introducing alternative wheat cultivars with early or late heading dates (−10 to +10 days from the heading date of the Geumgang cultivar).

Heading Date	Cultivar	Future Period	Incidence (%)	Percent Change in Incidence (%)
Before 10 days (-10)	Arijinheuk	2041–2071 2071–2100	4.9 6.8	-32.1 -26.1
Before 5 days (-5)	Baekgang, Hwanggeumal, Joeun, Jogyeong, Johan, Jojung, Jonong, Jopum, Saeol	2041–2071 2071–2100	6.0 7.9	-17.3 -14.7
0	Dabun, Geumgang, Jeokjung, Joa, Jungmo2008	2041–2071 2071–2100	7.2 9.2	0 0
After 5 days (+5)	Anbaek, Baekchal, Baekjung, Cheonggye, Dajung, Gobun, Goso, Hanbaek, Hojung, Jinpum, Milseong, Namhae, Ol, Olgeuru, Seodun, Sinmichal1, Suan, Sugang, Tapdong, Uju, Uri, Yeonbaek	2041–2071 2071–2100	8.6 10.9	19.5 18.1
After 10 days (+10)	Alchan, Dahong, Eunpa, Geuru, Saegeumgang, Sinmichal, Taejung	2041–2071	10.2	40.7
		2071–2100	12.9	39.7

**Figure 4:**
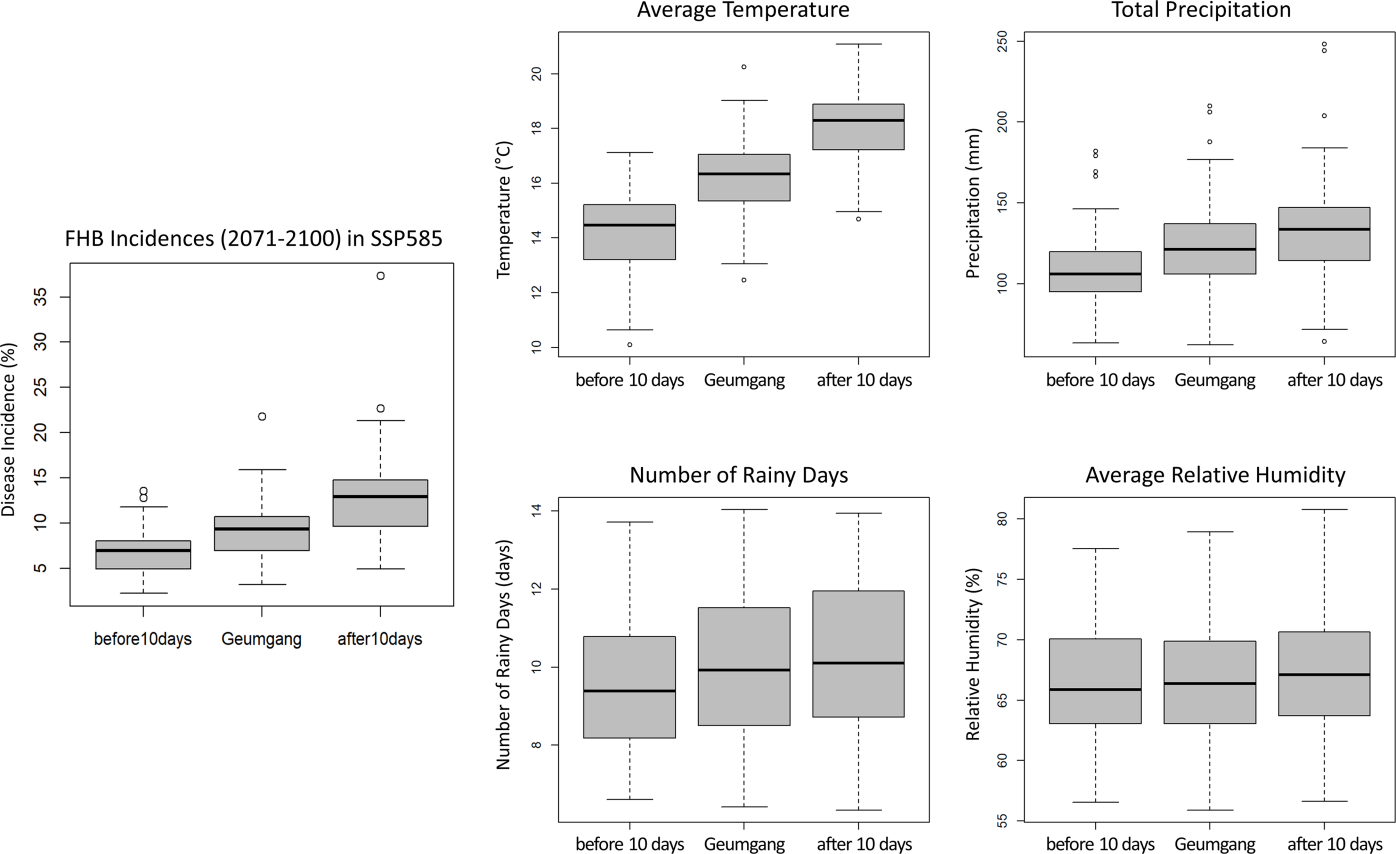
Comparisons of the adaptation strategy using early (before 10 days) or late (after 10 days) heading dates for the projected FHB incidences and the corresponding weather conditions of average air temperature, total precipitation, number of rainy days, and average relative humidity during the duration of GIBSIM simulations. Box plots were made with the results from multiple wheat-suitable areas based on the SSP585 scenario for the distant future period (2071-2100).

To understand the key weather variables affecting the projected changes in FHB incidences with varying heading dates, we extracted and analyzed the weather data used to simulate the GIBSIM (**Figure 4**). Graphical comparisons of the ranges of four weather variables (average air temperature, total precipitation, number of rainy days with more than 0.3 mm of precipitation, and average relative humidity) between the cultivars with different heading dates (before and after 10 days) indicated that the average air temperature and FHB incidences had the most similar box plot distribution. Thus, the temperature was the main factor that changed with a shift in the heading dates. Although the median values of the other three variables showed a similar increasing trend (from before to after 10 days) to the ones of FHB incidence, the heights of their box plots considerably overlapped, making the graphical interpretation of the FHB incidence and these weather variables interaction difficult.

Therefore, to further understand the relative contribution of individual weather variables, a regression analysis was conducted with FHB incidence as a dependent variable and four weather variables as an explanatory variable (**Supplementary Figure S4**). The linear regression results showed that with 0.83 of the coefficient of determination (R^2^), the average air temperature had a relatively pronounced positive effect (coefficient: +1.28) with a confidence level of 99%, followed by the average relative humidity with a positive effect (coefficient: +0.30) with a confidence level of 99%, and the number of rainy days with a positive effect (coefficients: +0.22) with a confidence level of 99%. In contrast, total precipitation showed a very minimal positive effect (coefficient: +0.004) with no statistical significance in the regression result. These results indicated that the changes in air temperature, relative humidity, and rainy days significantly affected the resulting FHB incidences when the heading date was changed. Overall, our findings suggest that adopting alternatives or breeding new cultivars with early heading dates can be an effective adaptation strategy to manage the FHB epidemics better under climate change conditions in the Korean Peninsula.

## 4 Discussion and conclusions

We performed a series of modeling studies on wheat phenology and FHB epidemics in response to climatic conditions and successfully evaluated the impacts of climate change by sequentially integrating the modeling results. The integrated modeling approach, combining all results of wheat suitability, heading dates, and FHB incidences, showed gradual but continuous increases in the FHB incidence towards 2100, with different temporal and spatial patterns of varying magnitudes depending on the two SSP scenarios. To counter the projected increases in FHB incidence in the Korean Peninsula, a practical adaptation strategy utilizing currently available wheat cultivars, either with earlier or later heading dates compared to the Geumgang cultivar used in the study, was investigated. Replacing the Geumgang cultivar with the ones with an earlier heading date resulted in a substantial reduction in FHB incidence in future periods.

### 4.1 Simulation of suitable areas and heading dates for winter wheat in the Korean Peninsula

Based on the optimal climatic conditions for wheat cultivation, we simulated the potential expansion of areas suitable for wheat cultivation under climate change. This suitability mapping for areas where wheat fields are likely to be located in the future is a prerequisite to making the subsequent FHB risk projections more realistic and reasonable. Nevertheless, current wheat cultivation areas in the Korean Peninsula may not be+ the same as the simulated suitable areas. This is because wheat cultivation in Korea involves many factors besides climate. Thus, in several instances, wheat is not grown even if the area is climatically suitable for cultivation. Owing to the recent COVID-19 pandemic, frequent conflicts between countries, and the resultant surge in international wheat price, the South Korean government has been trying to increase domestic wheat production by implementing the “wheat industry development basic plan” policy aimed at achieving a 10% wheat self-sufficiency rate by 2030. These ongoing efforts may encourage the expansion of wheat cultivation areas to the simulated suitable areas in the near future.

A potential but more realistic FHB risk projection under climate change was derived by linking the GIBSIM with the DVR model to realize the actual field interactions of wheat and FHB during the flowering period of wheat. In this study, the DVR model was adopted to predict the heading date of the Geumgang cultivar. The simulations of heading date using the DVR model resulted in an advancing trend over future periods, reflecting the temperature rise under climate change. The GCMs of the SSP245 and SSP585 scenarios used in this study predicted that global mean temperature would rise by 3.02°C and 5.20°C by 2100, respectively (Sung et al., 2021). Our results showed a reasonable shift in the wheat heading date affected by this projected temperature increase. Considering that the existing ecophysiological models were able to predict the heading date of wheat with good accuracy with root mean square errors as low as 4 to 7 days (Asseng et al., 1998; White et al., 2008; Zheng et al., 2013; Bogard et al., 2014), the performance of the DVR model (5.1 days of RMSE) used in this study is reasonably good in predicting the heading date of the Geumgang cultivar (Kim et al., 2022). Nevertheless, to apply the GIBSIM model to other cultivars currently grown in Korea, adopting a mechanistic phenology model, such as the Sirius model (Jamieson et al., 1998), for the prediction of heading date should be considered. This is because mechanistic models consider the interactions between environment and genotype, represented by ‘genetic parameters’ reflecting genetic variation among cultivars (Bogard et al., 2014).

### 4.2 Lack of the GIBSIM model calibration in the context of Korea

The regression equation between the simulated FHB risk indices of GIBSIM and the actual FHB incidences collected in the wheat fields of Korea showed that the GIBSIM simulations explain only 55% of the variation in the observed FHB incidences. Although similar levels of goodness-of-fit between the observed and simulated values is often found in many previous studies with plant disease or growth models (Burleigh et al., 1972; Hooker et al., 2002; Rossi et al., 2003; Liang et al., 2016; Jevtić et al., 2017), further effort should be made to improve the model performance. There are three possible reasons for the relatively low correlation between the observed FHB incidences and GIB% from the GIBSIM. First, some of the parameters and embedded algorithms of the GIBSIM are empirically developed based on the observations in Brazil (Del Ponte et al., 2005). This indicates these original parameters and algorithms may need to be calibrated or modified, respectively, to reflect the local-specific characteristics of the environment, pathogen strains, and wheat cultivars in Korea. In fact, this kind of model localization is required to minimize uncertainty and generate optimal model performance when adopting an existing model developed in other countries or regions (Andrade-Piedra et al., 2005; Kim KH et al., 2018; Rotter et al., 2012). Secondly, the quality of the observed FHB incidence data was low, as the spatial resolution of the data was low at the district (*si* or *gun* in Korea) level. Lastly, the corresponding weather data to run GIBSIM were obtained from the nearest ASOS weather station; thus, they might not represent the local weather conditions where the FHB incidence data were collected. To support the calibration study of GIBSIM in a future study, the corresponding metadata, such as coordinates and data collection dates, should be collected and used with the FHB incidence data (Donatelli et al., 2017). Location-specific, long-term, FHB occurrence data should be systematically collected, with the corresponding metadata, to enable further statistical modeling (Lee et al., 2022a) or process-based model calibration and validation (Kim KH et al., 2018). High-resolution grid products, such as the 1-km interpolated weather observation data from the KMA (https://data.kma.go.kr), need to be evaluated for the accuracy of the weather variables needed for the GIBSIM simulation. Although weather conditions largely determine the occurrence of FHB, agronomic factors also significantly influence FHB. Potential factors to be considered in future modeling efforts with GIBSIM include fungicide treatment, crop rotation system (with rice), weed management, and host plant resistance in Korea (Schaafsma and Hooker, 2007; Landschoot et al., 2011).

### 4.3 Adaptation strategies to the projected impacts of FHB in the Korean Peninsula

Impact assessments of FHB epidemics in the future indicate that the incidence of FHB in the Korean Peninsula is projected to increase in the future, and the projected incidence of FHB is slightly higher in North Korea than that in South Korea. Graphical comparisons for four essential weather variables between North and South Korea showed that air temperature and relative humidity were higher (favorable to FHB epidemics) in North Korea (**Supplementary Figure S5**). Wheat-suitable areas have expanded northward, covering much of the North Korean area in the distant future, indicating that more wheat cultivation may be possible in North Korea. Because of the economic sanctions and border closures due to COVID-19 over the last few years, North Korea is aggressively promoting wheat cultivation to fight for food security these days (news articles not shown). Considering the inevitable vulnerability of wheat cultivation due to the rapid expansion of wheat cultivation and the projected increase of FHB epidemics from this study, proactive, adaptive measures, supported by international humanitarian efforts supplying diverse wheat cultivars and essential agricultural inputs, should be prepared prior to actual cultivation of wheat in North Korea.

A possible adaptation strategy to the projected epidemics of FHB is suggested in this study, which uses alternative wheat cultivars with earlier heading dates compared with the Geumgang cultivar. Changing planting dates or using cultivars with different maturity to avoid the high disease pressure period is one of the most popular adaptation strategies to mitigate the projected impacts of climate change on plant diseases. In the study, wheat cultivars with 10 days earlier heading date showed a larger reduction in FHB incidences compared to those with 5 days earlier heading dates. However, cultivars with 10 days earlier heading dates, such as Arijinheuk (**Table 2**), have very limited availability on the market. However, the Baekgang, Hwanggeumal, Joeun, Jogyeong, Johan, Jojung, Jonong, Jopum, and Saeol cultivars, with 5 days earlier heading date, can also be an alternative as they reduce the projected risk for the near future period by 17.3%. Nevertheless, applying this adaptation strategy is not simple, as there are other factors to consider selecting alternative cultivars, such as stress tolerance, length of maturity, socioeconomic factor, and preference in the market. In addition, the assumption of a phenophase date for a plant is difficult to apply to annual crops, such as rice and wheat, which are more significantly affected by human-dependent planting and cultivation activities. In Korea, wheat is generally grown in crop rotation with rice, wherein winter wheat is grown after the rice harvest. Therefore, the possible period of wheat growth should not interfere with the rice cultivation period. Considering these potential conflicts, an integrated modeling solution that includes both wheat and rice growth models should be developed. Overall, these indicate that reasonable adaptation strategies are difficult to implement even if they are properly presented.

### 4.4 Management of uncertainty in the climate change impact and adaptation study and the way forward

In addition to the projected FHB epidemics using only climatic factors, we expect that the epidemics of FHB will be more complicated in the future because of the complex interaction of climatic, genetic, and agronomic factors. This indicates that continuous studies are needed to get a clearer picture of uncertain climate change and the resulting disease risks in Korea. First, reducing the uncertainty level by removing or quantifying uncertain factors and better understanding used-to-be uncertain factors must be pursued through active research and development. For example, using 18 GCMs for each SSP scenario compensates for some systematic uncertainty inherent in GCMs (Mathukumalli et al., 2016; Ouma et al., 2016). Further, uncertainty in individual ecophysiological models, which leads to low reliability of the model, must be addressed. As mentioned previously, the main sources of model uncertainty include insufficient integration of non-climatic confounding factors in modeling and poor calibration and validation due to limited ground-truth data. As more location-specific ground-truth data are obtained through long-term and regular surveys, more reliable models can be used for impact studies. In addition, significant uncertainties account for all factors influencing plant disease epidemics, some of which cannot be predicted with the currently available knowledge and techniques. Various environmental factors not directly considered in the model, such as elevated CO_2_ levels, soil nutrients, and other weather variables, can also influence future ecophysiological interactions (Velásquez et al., 2018). Therefore, a comprehensive process-based and integrated modeling approach that includes the ecophysiological responses of a plant, its interactions with pathogens, and environmental factors may be considered for future studies.

In this study, we presented an impact assessment and adaptation study of wheat FHB epidemics using an integrated modeling approach with multiple models. To alleviate projected increases in the FHB epidemic over the next 30 to 60 years, we used an integrated modeling solution to investigate a scientifically informed, long-term adaptation strategy replacing the original cultivar with a disease-resistant cultivar or a cultivar that can avoid high disease pressure by shortening the duration before the heading date. In addition, the impact assessment in each administrative district of the Korean Peninsula also needs to develop localized adaptation strategies for local government units in the local context. For example, an early warning system using short-term (2–3 days) to mid-term (7–15 days) weather forecasts and an FHB infection model would be useful for wheat growers to implement timely preventive controls of FHB. As this type of integrated modeling platform is developed and continuously improved with more quality-controlled data and more reliable modeling algorithms, policymakers and agricultural stakeholders will be able to prepare more realistic, rational adaptation strategies to deal with the upcoming climate change threat based on evidence-based scientific results. Many follow-up studies are needed in the near future, as indicated by the findings of this study.

## Data availability statement

The original contributions presented in the study are included in the article/**Supplementary Material**. Further inquiries can be directed to the corresponding author.

## Author contributions

J-YJ, J-HK, K-HK conceived and initiated the study and led the manuscript preparation. CC, JC collected and managed the required data for the analysis. J-YJ, J-HK, MB, JK conducted modelling and analysed the results. WP sourced the GIBSIM and provided advice and guidance for the study. All authors contributed to the article and approved the submitted version.
