# Supplementary material for: Adapting to the projected epidemics of Fusarium head blight of wheat in Korea under climate change scenarios
Source: Front Plant Sci. 2022 Dec 9;13:1040752. doi: 10.3389/fpls.2022.1040752 (PMC9793406; doi:10.3389/fpls.2022.1040752)
Supplement: Supplementary file 1 [file DataSheet_1.docx]

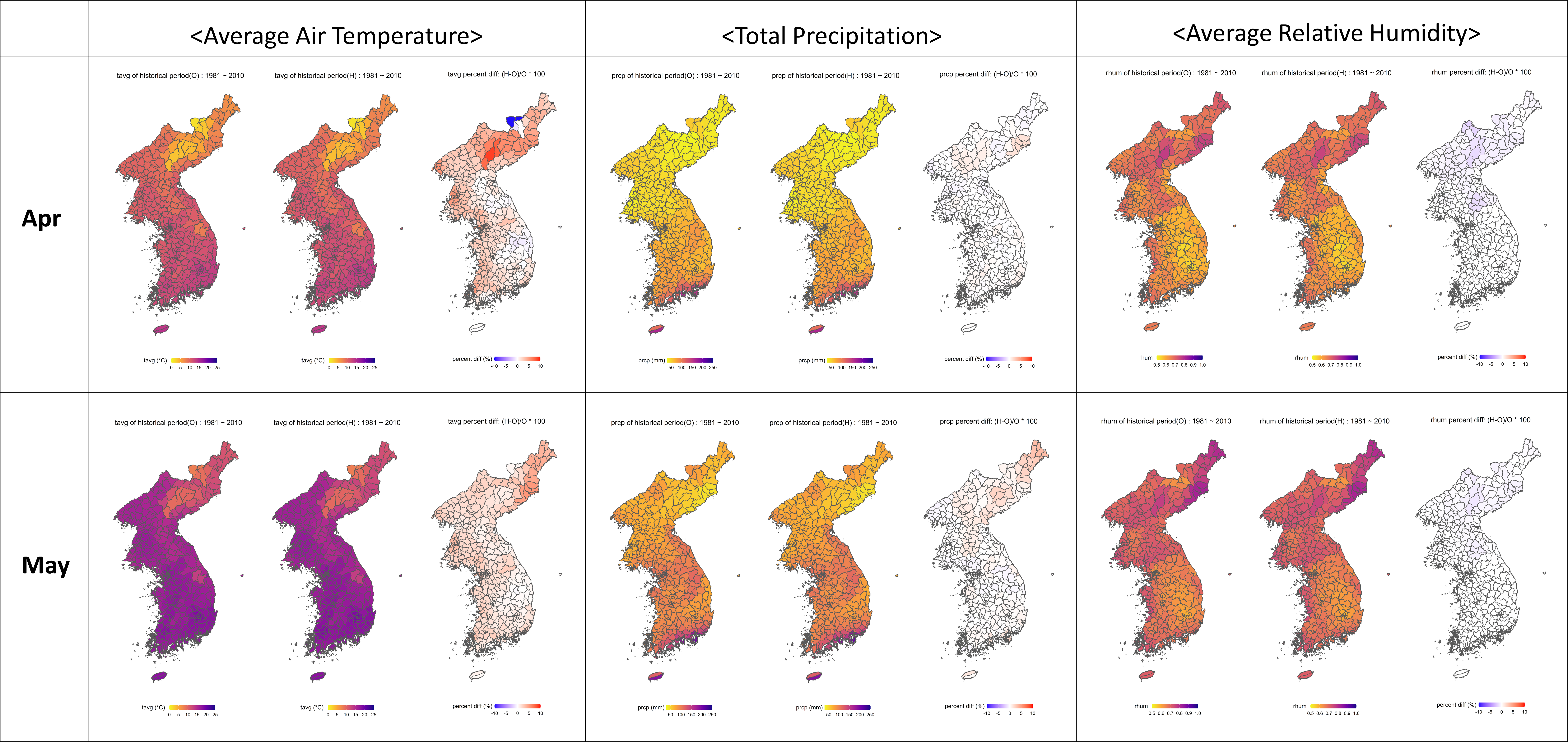


Figure S1. Reproducibility test results with the bias-corrected and downscaled CMIP6 scenarios from 18 GCMs (H) compared with the ASOS observed weather data (O). Reproducibility test was conducted for three weather variables (average air temperature, total precipitation, average relative humidity) for April and May for the historical period (1981-2010). Note that percent differences (Percent diff) between the scenario and observed weather data were visualized on the map.
