# Supplementary material for: Adapting to the projected epidemics of Fusarium head blight of wheat in Korea under climate change scenarios
Source: Front Plant Sci. 2022 Dec 9;13:1040752. doi: 10.3389/fpls.2022.1040752 (PMC9793406; doi:10.3389/fpls.2022.1040752)
Supplement: Supplementary file 2 [file DataSheet_2.docx]

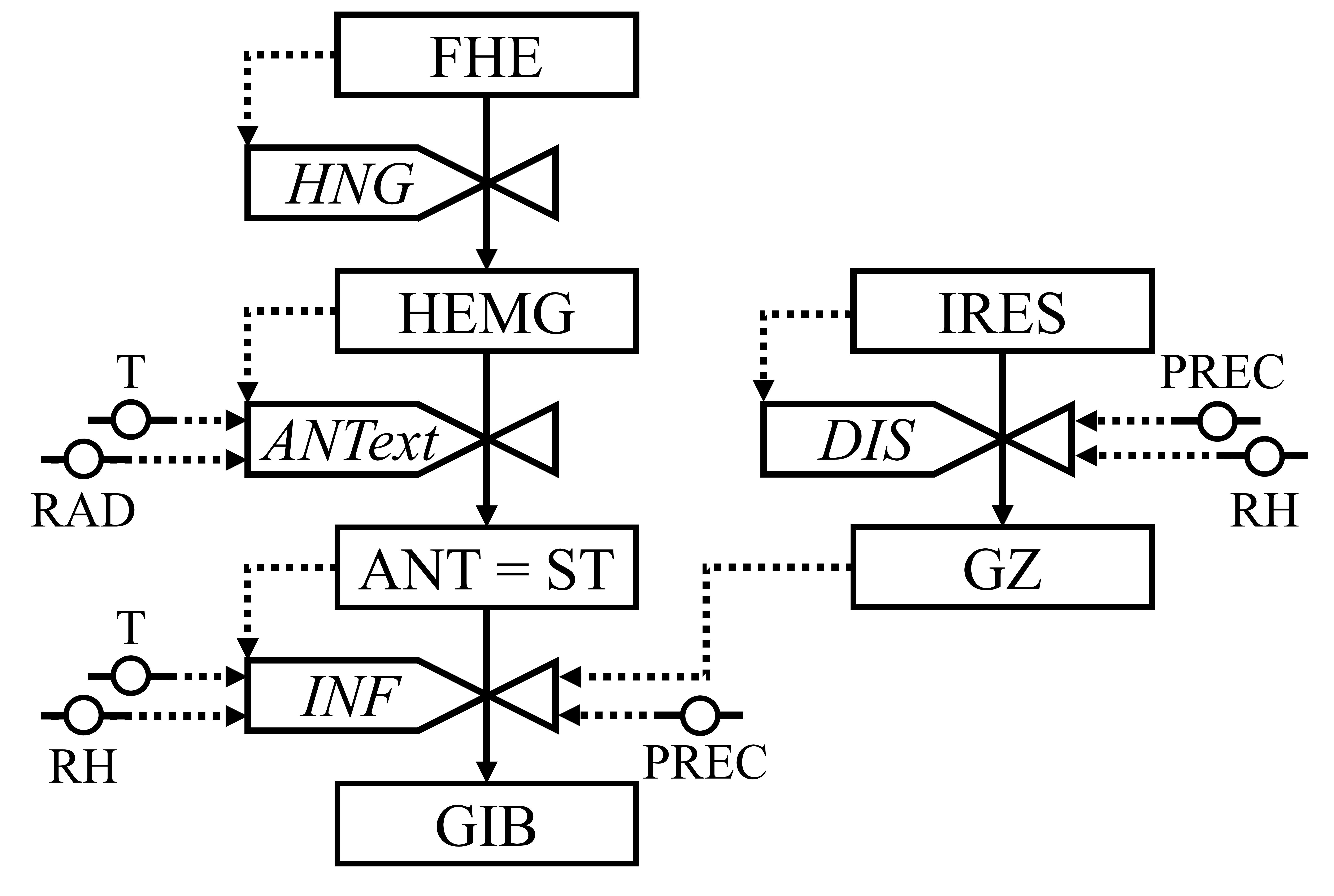
Diagram for the modeling structure of the GIBSIM

Figure S2. Diagram for the modeling structure of the GIBSIM (Adopted from FIG. 1 in Del Ponte et al. (2005)). Abbreviations: FHE = First heads emerged; HNG = daily heading rate; HEMG = proportion of heads emerged; ANText = daily anther’s extrusion rate; ANT = daily proportion of anthers present; ST = proportion of susceptible tissue based on ANT and coefficients for susceptibility after peak flowering up to 14 days after flowering ends; INF = daily infection frequency; IRES = Inoculum present on crop residues; DIS = daily inoculum dispersal rate; GZ= relative density of a spore cloud; GIB = daily risk infection index; T =daily mean temperature (°C); RAD = daily solar radiation (MJ/m2); RH = daily mean relative humidity (%) and PREC = daily precipitation (mm).

Simulation mechanisms and equations

The model simulation was initiated when the first head fully emerged in the field (FHE), which lasts more than 30 days in general until susceptible tissues no longer exist for a new infection as the model consider post-flowering infection. In the model, FHB infection is generally associated with anthers, where fungal spores land and grow into kernels, glumes, or other spike tissues (Sutton, 1982; McMullen et al., 1997). In the simulation process for FHB infection, the daily proportion of heads that emerged (HEMG) was first determined as a function of heading rate (HNG) (Eq. S1). HNG is the daily cumulative proportion of heads that emerged in a 1-meter section of the wheat plot.

(S1)

$$HNG=1-\exp(-0.0127 t^{2.4352})$$

where: *t* = 1 day.

The daily proportion of extruded anthers (ANT), acting as susceptible tissue (ST) to new infection, was calculated using the anthers’ extrusion rate (ANText) (Eq. S2). ANText is the daily rate of cumulative proportion of extruded anthers in a cohort of heads. ANText was applied independently to each head.

(S2)

$$ANText=1-\exp(a t^{b})$$

where *t* = 1 day; *a* = 0.255 − 0.029T + 0.0009T^2^; b= −5.773 + 0.966T −0.0278T^2^.

Anther extrusion changes depending on several conditions. The first anther of each head extrudes three days after head emergence. The longevity of extruded anthers is determined to be in the range of 2 to 5 days, depending on daily solar radiation conditions. Here, the host factor is represented by ST, the proportion of susceptible tissue, which is based on ANT, and the coefficients for susceptibility after peak flowering up to 14 days after flowering ends.

The inoculum was assumed to be present in the residues (IRES). The inoculum factor is represented by GZ and daily relative density of an airborne FHB spore cloud. GZ is a function of the daily mean relative humidity and CRD (a dummy variable for consecutive rainy days) (Eq. S3).

(S3)

$$GZ={(-0.6306+0.0152 RH+0.1076 CRD)}^{2}$$

where RH = daily mean relative humidity (%) and CRD = dummy variable for the position of a rainy day in a consecutive period of rainy days.

Whether FHB infection occurs is determined based on the combination of rainfall and relative humidity in a two-day window. If infection occurs, INF, the proportion of susceptible tissue likely to be infected at any time, is calculated using an exponential model with the average mean daily temperature (Eq. S4).

(S4)

$$INF=0.001029 exp(0.1957 T)$$

where T = average mean daily temperature in the two-day window of the infection event

Finally, the daily infection risk index (GIB) is the product of the proportion of susceptible tissue (ST), infection frequency (INF), and FHB conidial cloud density (GZ). The final results of the model were obtained by combining these three factors using Eq. (S5).

(S5)

$$GIB\%=\sum(ST*INF*GZ*100)$$

where ST is the mean daily proportion of susceptible tissue during a two-day infection event (IE), INF is the infection frequency on the second day of IE, GZ is the mean FHB spore cloud density during IE, and GIB% is the accumulated GIB percentage value.

**References**

Del Ponte, E. M., Fernandes, J. M. C., and Pavan, W. (2005). A risk infection simulation model for fusarium head blight of wheat. *Fitopatologia Brasileira* 30(6), 634–642. doi: 10.1590/S0100-41582005000600011

McMullen, M., Jones, R., and Gallenberg, D. (1997). Scab of Wheat and Barley: A Re-emerging Disease of Devastating Impact. *Plant Dis*. 81(12), 1340–1348. doi: 10.1094/PDIS.1997.81.12.1340

Sutton, J. C. (1982). Epidemiology of wheat head blight and maize ear rot caused by Fusarium graminearum. *Can. J. Plant. Pathol*. 4(2), 195-209. doi: 10.1080/07060668209501326
