# Supplementary material for: Adapting to the projected epidemics of Fusarium head blight of wheat in Korea under climate change scenarios
Source: Front Plant Sci. 2022 Dec 9;13:1040752. doi: 10.3389/fpls.2022.1040752 (PMC9793406; doi:10.3389/fpls.2022.1040752)
Supplement: Supplementary file 3 [file DataSheet_3.docx]

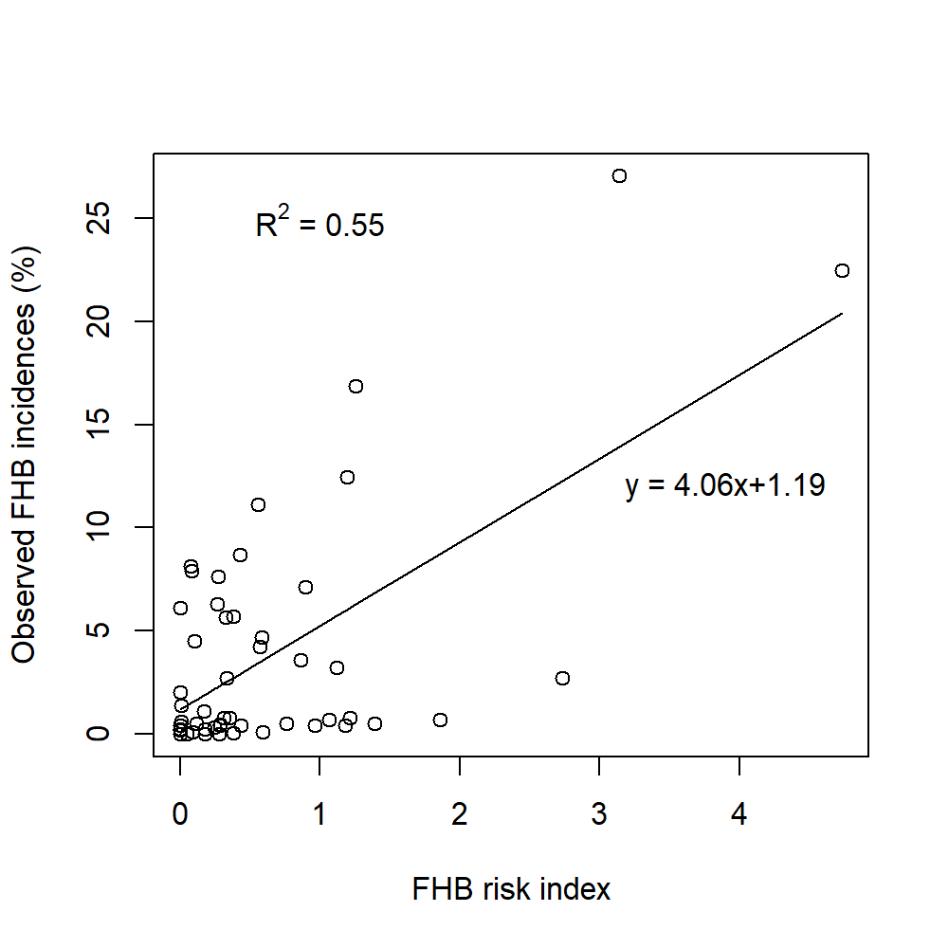
Figure S3. Regression between the simulated FHB risk index (GIB%) from the GIBSIM and the observed FHB incidences (N=52) collected from the actual fields.
