# Supplementary material for: Adapting to the projected epidemics of Fusarium head blight of wheat in Korea under climate change scenarios
Source: Front Plant Sci. 2022 Dec 9;13:1040752. doi: 10.3389/fpls.2022.1040752 (PMC9793406; doi:10.3389/fpls.2022.1040752)
Supplement: Supplementary file 4 [file DataSheet_4.docx]

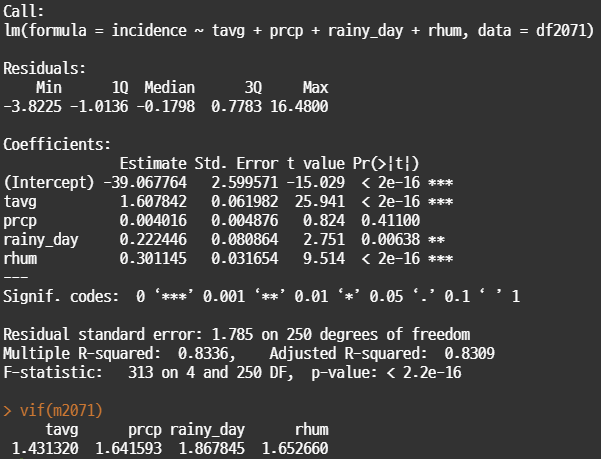


Figure S4. Linear regression result with the FHB incidence as a dependent variable and the four weather variables data as an explanatory variable. Abbreviations, tavg: average air temperature, prcp: total precipitation, rainy_day: number of rainy days with more than 0.3 mm of precipitation, and rhum: average relative humidity.
