# Supplementary material for: Adapting to the projected epidemics of Fusarium head blight of wheat in Korea under climate change scenarios
Source: Front Plant Sci. 2022 Dec 9;13:1040752. doi: 10.3389/fpls.2022.1040752 (PMC9793406; doi:10.3389/fpls.2022.1040752)
Supplement: Supplementary file 5 [file DataSheet_5.docx]

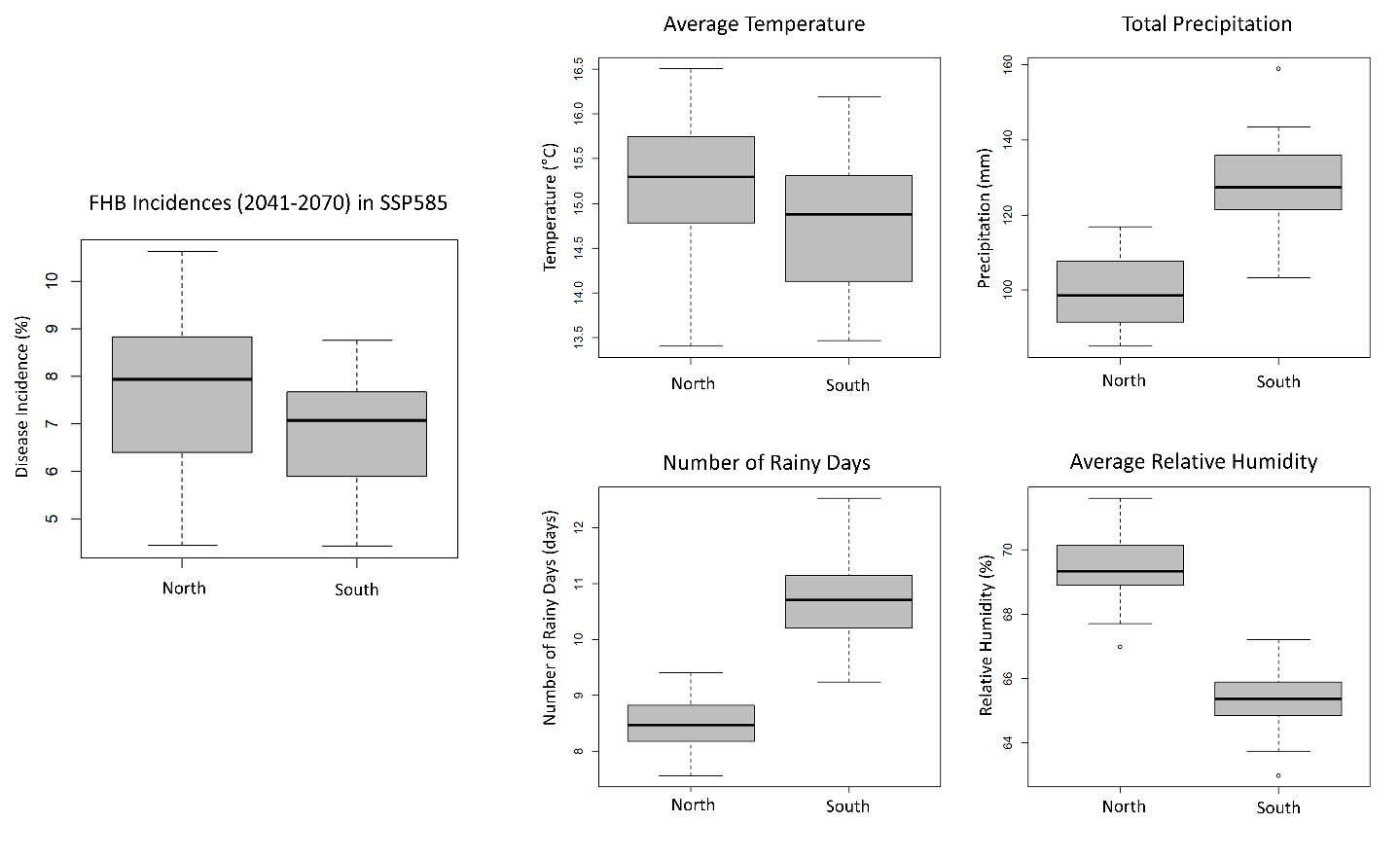


Figure S5. Comparisons of the potential FHB incidences for the North and South Korean regions and the corresponding weather conditions of average air temperature, total precipitation, number of rainy days, and average relative humidity during the duration of GIBSIM simulations. Box plots were made with the results from multiple wheat suitable areas based on the SSP585 scenario for the near future period (2041-2070).
